# Entanglement transmission due to the Dzyaloshinskii–Moriya interaction

**DOI:** 10.1038/s41598-023-29995-x

**Published:** 2023-02-20

**Authors:** Mostafa Motamedifar, Fatemeh Sadeghi, Mojtaba Golshani

**Affiliations:** Faculty of Physics, Shahid Bahonar University of Kerman, Kerman, 7616913439 Iran

**Keywords:** Physics, Quantum physics

## Abstract

We revisited the effectiveness of state and entanglement transmission through a spin-chain-based quantum channel while altering the system parameters and the channel’s initial state. Our research is focused on the spin-1/2 XX chain with Dzyaloshinskii–Moriya (DM) interaction and the aim is to measure entanglement dynamics between different part of the chain. The speed of entanglement propagation is utilized to probe the evolution of the system via three scenarios: (i) pure Heisenberg interaction, (ii) pure DM interaction, and (iii) collaboration of both types of couplings. To accomplish this, we employ the fermionization approach to obtain an exact solution to the problem. Aside from investigating the influence of magnetic interaction type on entanglement transfer, the effect of selecting the initial state has also been studied. As a result, we discovered that the phase factor regulating the system’s initial state induces sharp drops in the propagation speed of entanglement. We also showed how to predict the location of these dramatic drops using the language of wave interference. In addition, the fastest transmission occurs at a special value of the phase factor in which the highest amount of entanglement reaches the system’s different pairs. On the other hand, we observe a continuous and flat range of this factor in which the least amount of entanglement is transmitted and for them we have a sharp drop in the speed profile.

## Introduction

There are several platforms that might be used to implement quantum computation^1–3^, such as cold atoms^4^, trapped ions^5^ and superconducting quantum circuits^6^. One of the most important needs for such systems is the faithful and fast quantum state transfer (QST). Despite the fact that long-distance quantum communication has been extensively established in optical fibers^7^, it is still essential to discover a viable method for transmitting quantum states using solid-state devices. Alongside various solid-state materials suggested for performing QST protocols, spin chains have received a great deal of interest^8–15^, and have supported numerous technologies already being developed.

Bose demonstrated in Ref.^8^ that an unmodulated Heisenberg spin chain may function as a quantum data bus over realistic distances. The communication is accomplished by encoding a quantum state on a spin at one end of the chain, then waiting for an amount of time, to get to the other end of the chain. Alongside making use of spin chains, Christandl et al., revealed in Ref.^9^ that by modulating the coupling strengths between the chain’s spins, the fidelity of quantum communication may be considerably increased. A significant benefit of the mentioned strategies is that state transfer happens due to the interaction of the chain’s spins, and no dynamical control is necessary. Proposals to realize this technique cover a wide range of usage from superconducting nanocircuits to solid-state systems.

On the other hand, in a time-dependent scenario of QST, the exchange coupling between spins are dynamically adjusted throughout the evolution. The most obvious procedure in this case is to employ a sequence of swap operations between nearby sites to progressively move state along the chain, while different representative strategies are explored in Refs.^16–19^. A major class of such a scenario falls into the adiabatic process, while the most of them are restricted to small number of qubits.

To get over the limitation imposed by the number of particles, we illustrate how a QST operates in momentum space to analyze the state transfer in a many-body system. Furthermore, by figuring out the speed of entanglement propagation we answer a key question: how fast does a QST happen? In fact, we employed the entanglement concepts to monitor the QST process when the state transfer is supposed to be accompanied by the entanglement propagation. Working in the framework of entanglement to reveal state transitions is a highly popular and efficient method^20,21^ just to name a few. In the core idea of the present work, we proposed that an entangled state, encoded on the qubits at beginning of the system, is supposed to be transmitted to the other pairs of the chain. When we mention chain, we mean the XX model with added Dzyaloshinskii–Moriya (DM) interaction. The featured effects of DM interaction on entanglement dynamics in finite size systems have been examined in Refs.^22,23^.

Beyond two aforementioned methods i.e., modulating chain and dynamically tuning exchanges, there is another approach to increasing the transmission speed. In the present work, we disclose how adding DM interaction between the nearest sites of a chain does affect on propagation speed. Our work goes beyond Ref.^24^ by proposing all possible scenarios about magnetic interactions. With the alternative analytical approach we utilized, it is discovered that in several subjects, our findings disagree with those reported in Ref.^24^, but in the others, we have excellent agreement. For example, we can mention the sudden drops in the speed profile triggered by specific values of the phase factor that regulates the system’s initial state. This dilemma imposes additional outcomes to occur. For instance, in the presence of both types of magnetic interactions (DM and Heisenberg), the maximum amount of entanglement that reaches various pairs depends on the value of magnetic interactions (contrary to what was stated in Ref.^24^). The fidelity function has several disagreements in its behavior as well. Of course, since the analytical approach used here ends in the derivation of a closed form for the wave function, it is assumed that more accurate results have been attained in this work.

For dealing with the above situations in details, we organized the manuscript as follows: Section “The model and state evolution” introduces the XX chain with extra DM interaction, as well as the essential stages of analytical methodologies employed to address it. Section “Method” explains the measure used in this work for quantifying entanglement. The most interesting results for the entanglement propagation is discussed in Section “Results”. Finally Section “Conclusion” summarizes conclusions and outlooks.

## The model and state evolution

To analyze the influence of magnetic interaction on entanglement evolution of the spin-1/2 Heisenberg chain, we consider the Hamiltonian

$$\begin{aligned} {{\mathcal {H}}}= {{\mathcal {H}}_{XX}} + {{\mathcal {H}}}_{DM} \,, \end{aligned}$$

where

1 $$\begin{aligned}{} & {} {{\mathcal {H}}_{XX}} = \sum _{j}\left[ J(S^{x}_{j}S^{x}_{j+1}+S^{y}_{j}S^{y}_{j+1})\right] , \end{aligned}$$

is the Hamiltonian of the Heisenberg XX chain and ${{\mathcal {H}}}_{DM}$ is the DM term provided in

2 $$\begin{aligned} {{\mathcal {H}}}_{DM}=\sum _{j}\vec {D}\cdot [\vec {S}_{j}\times \vec {S}_{j+1}]. \end{aligned}$$

In the following, we assume that $\vec {D}$ is aligned in the ${\hat{z}}$ direction of spin space i.e., $\vec {D} =(0,0,D)$. Moreover, making use of the Jordan-Wigner transforms:

3 $$\begin{aligned} S_{j}^{+}= & {} c^{\dagger }_{j} \exp \left( i\pi \sum _{i< j} c^{\dagger }_{i}c_{i}\right) \,,\nonumber \\ \quad S_{j}^{-}= & {} \exp \left( -i\pi \sum _{i < j}c^{\dagger }_{i}c_{i}\right) c_{j}\,,\nonumber \\ S^{z}_{j}= & {} c^{\dagger }_{j}c_{j} -1/2, \end{aligned}$$

We recast the original lattice spin Hamiltonian in the context of spinless fermions as follows:

4 $$\begin{aligned} {{\mathscr {H}}}_{f}=\sum _{j}\Bigg (\frac{(J+iD)}{2} c^{\dagger }_{j} c_{j+1}-\frac{(J-iD)}{2}c_{j}c^{\dagger }_{j+1}\Bigg ), \end{aligned}$$

where $c^{\dagger }_{j}$ ($c_{j}$) is the spinless fermion creation (annihilation) operator on site *j*. Consequently, using Fourier transformation of the form:

5 $$\begin{aligned} c_j =\frac{1}{\sqrt{N}}\sum _k \, {\tilde{c}}_k e^{-ikj}, \end{aligned}$$

the diagonalized Hamiltonian in the momentum space is given by

6 $$\begin{aligned} { \mathscr {H}_f} =\sum _k \, \Big (\underbrace{J \cos (k)+D \sin (k)}_{\epsilon (k)}\Big ){\tilde{c}}^{\dagger }_{k} {\tilde{c}}_{k}. \end{aligned}$$

In what follows, we will demonstrate how an initially entangled state between two first neighbors of the chain propagates across that. Consider two neighbors (*A*, *B*) that are initially in an entangled state while the remainder of the system is initially separable. Then the initial quantum state of the system reads:

7 $$\begin{aligned} |\psi (t=0)\rangle = \frac{|1_A 0_B\rangle +e^{i\phi }|0_A 1_B\rangle }{\sqrt{2}}\otimes |\underbrace{00..0}_{N-2}\rangle . \end{aligned}$$

Here, *N* is the number of the system’s particles and $|0\rangle$ $(|1\rangle )$ represents the eigenstate of the $S^z$ operator with eigenvalue of $-1/2$ (1/2) and $\phi$ is an arbitrary phase factor. In what follows, we select the first two qubits of the chain as the pair of the form (*A*, *B*). Consequently, for arriving time evolution of the system’s quantum state we make use of $|\psi (t)\rangle =U(t)|\psi (0)\rangle$, where $U(t)=exp(-i t{\mathcal {H}})$ is the time evolution operator and $\hbar \equiv 1$. Then, using Eq. (4) we have $|\psi (t)\rangle$ in the fermionic language:

8 $$\begin{aligned} e^{-i t\mathcal {H}}|\psi (0)\rangle \longrightarrow \frac{e^{-i t {\mathscr {H}}_f}}{\sqrt{2}} \big (c_A^\dagger +e^{i\phi }c_B^\dagger \big )|0\rangle , \end{aligned}$$

Plugging back into Eq. (5) and according to the position of qubits *A* and *B*, we arrive

9 $$\begin{aligned} |\psi (t)\rangle = \frac{e^{-i t \mathscr {H}_f}}{\sqrt{2N}} \big (\underbrace{\sum _k e^{ik}{\tilde{c}}_k^\dagger }_{c_A^\dagger }+e^{i\phi }\underbrace{\sum _k e^{i2k}{\tilde{c}}_k^\dagger }_{c_B^\dagger }\big )|0\rangle . \end{aligned}$$

Returning to Fourier form of Hamiltonian [Eq. (6)] and inserting it in Eq. (9) we obtain

10 $$\begin{aligned} |\psi (t)\rangle = \frac{e^{-i t\sum _q \epsilon (q) {\tilde{c}}^{\dagger }_{q} {\tilde{c}}_{q}}}{\sqrt{2N}} \sum _k \big (e^{ik}+ e^{i(2k+\phi )}\big ){\tilde{c}}_k^\dagger |0\rangle . \end{aligned}$$

A series solution for the unitary time evolution operator in the same space can be given by

11 $$\begin{aligned} e^{-i t\sum _q \epsilon (q) {\tilde{c}}^{\dagger }_{q} {\tilde{c}}_{q}}=1-it\sum _q \epsilon (q) {\tilde{c}}^{\dagger }_{q} {\tilde{c}}_{q}-\frac{t^2}{2!}\sum _{q',q}\epsilon (q')\epsilon (q){\tilde{c}}^{\dagger }_{q'} {\tilde{c}}_{q'}{\tilde{c}}^{\dagger }_{q} {\tilde{c}}_{q}+..., \end{aligned}$$

by substituting Eq. (11) into Eq. (10) and taking into account $\{{\tilde{c}}_{q},{\tilde{c}}^{\dagger }_{k}\}=\delta _{qk}$ and ${\tilde{c}}^{\dagger }_{q}{\tilde{c}}_{q}{\tilde{c}}^{\dagger }_{k}|0\rangle =\delta _{kq}{\tilde{c}}^{\dagger }_{k}|0\rangle$ consequently we get

12 $$\begin{aligned} |\psi (t)\rangle = \frac{1}{\sqrt{2N}}\sum _k \Big [{\tilde{c}}^{\dagger }_{k}-it\epsilon (k) {\tilde{c}}^{\dagger }_{k} +\frac{(-it\epsilon (k))^2}{2!}{\tilde{c}}^{\dagger }_{k}+ \ldots \Big ] \big (e^{ik}+ e^{i(2k+\phi )}\big )|0\rangle , \end{aligned}$$

for $|\psi (t)\rangle$. Given that the ket of the initial state is defined in the spin space, we will accurately retrieve the coefficient of $|0\rangle$ back to the same space to ensure that our formalism lives in the correct space. So we replace ${\tilde{c}}_k$ with $c_j$:

13 $$\begin{aligned} |\psi (t)\rangle= & {} \frac{1}{\sqrt{2N}}\sum _k \big (e^{ik}+ e^{i(2k+\phi )}\big ) e^{-it\epsilon (k)}{\tilde{c}}^{\dagger }_{k} |0\rangle \biggr |_{\begin{array}{c} {\tilde{c}}^{\dagger }_{k}=\frac{1}{\sqrt{N}}\sum _j e^{-ijk}c^\dagger _j \end{array}}\nonumber \\= & {} \frac{1}{\sqrt{2}N}\sum _k e^{-it\epsilon (k)} \sum _j \big (e^{i(1-j)k}+ e^{i\phi }e^{i(2-j)k}\big )c^\dagger _j|00..0\rangle . \end{aligned}$$

Return to Eq. (6), $\epsilon (k)=J \cos (k)+D \sin (k)$, we can write $\epsilon (k) ={\tilde{J}}\cos (k-\alpha )$ where ${\tilde{J}}=\sqrt{J^2+D^2}$ and $\tan (\alpha )=D/J$. Then we reach

14 $$\begin{aligned} |\psi (t)\rangle =\frac{1}{\sqrt{2}N}\sum _k e^{-it{\tilde{J}}\cos \left( k-\alpha \right) } \sum _j \big (e^{i(1-j)k}+ e^{i\phi }e^{i(2-j)k}\big )c^\dagger _j|00..0\rangle . \end{aligned}$$

Since $\sum _k \big (e^{i(1-j+s)k}\big )= N\delta _{s,j-1}$ and $\sum _k \big (e^{i(2-j+s)k}\big )= N\delta _{s,j-2}$ then we can conclude that Eq. (14) becomes

15 $$\begin{aligned} |\psi (t)\rangle =\sum _j \underbrace{\frac{\Big (e^{-i(\frac{\pi }{2}+\alpha )(j-1)}\Big )}{\sqrt{2}}\Bigl [\mathcal {J}_{j-1}({\tilde{J}} t) +\mathcal {J}_{j-2}({\tilde{J}} t)e^{i(\frac{\pi }{2}+\alpha +\phi )}\Bigr ]}_{a_{j}(t)} c^\dagger _j|00..0\rangle , \end{aligned}$$

where $\mathcal {J}_s$ is the Bessel function of the first kind of order *s*^25^. In this equation, $a_j(t)$ is the probability amplitude of a fermionic particle occupying the $j^{th}$ site. The evolving wave function of the system employing single-particle states $\left( c^\dagger _j|00..0\rangle \right)$ is clearly described by Eq. (15). The $|\psi (t)\rangle$ is used to receive the system’s density matrix in any time, enabling us to establish a well-known entanglement quantifier, namely concurrence, for monitoring the system’s dynamical behavior in the next sections.

## Method

Quantum entanglement is a physical phenomenon that emerges when the quantum state of each particle of a group cannot be described autonomously from the state of the others.

In the present work, in order to investigate the transmission of entanglement across the chain, we must measure the amount of such quantity between nearest neighbor sites. That is, we examine how entanglement is transferred from one pair to another, and these pairs consist of two qubits that are the nearest neighbors. So throughout this article, wherever we mention the pair, we mean the two nearest neighbor qubits. One of the important quantifier of entanglement for two-qubit system is concurrence, introduced in Ref.^26^, which is applicable for both pure and mixed states. In Ref.^27^, concurrence is defined due to the reduced density matrix $\rho _{mn}$ of two qubits *m* and *n*. In fact, $\rho _{mn}$ can be obtained by tracing over all sites expect those at sites *m* and *n*, i.e., $\rho _{mn} = {\text {Tr}}_{all-(n,m)}(\rho )$, that can be expressed by two-point correlations as following fashion:

16 $$\begin{aligned} \rho _{mn}=\frac{1}{4} \sum _{\alpha , \beta =0}^{3} p_{\alpha \beta }~\sigma _{m}^{\alpha } \otimes \sigma _{n}^{\beta }, \end{aligned}$$

where $\sigma ^0$ is the $2\times 2$ identity matrix ($\mathbbm {1}$) and $\alpha =1,2,3$ addresses $\sigma ^x, \sigma ^y, \sigma ^z$ (Pauli matrices). Furthermore, because ${\hbox {tr}}\left( (\sigma _{m}^{\alpha } \otimes \sigma _{n}^{\beta }) \cdot (\sigma _{m}^{\alpha '} \otimes \sigma _{n}^{\beta '})\right) =4~\delta _{\alpha ,\alpha '}\delta _{\beta ,\beta '}$, then $p_{\alpha \beta }$ given by:

17 $$\begin{aligned} p_{\alpha \beta }={\text {Tr}}\left( \sigma _{m}^{\alpha }\otimes \sigma _{n}^{\beta } \rho _{m n}\right) =\left\langle \sigma _{m}^{\alpha }\otimes \sigma _{n}^{\beta }\right\rangle . \end{aligned}$$

Therefore, Eq. (16) can be written by matrix form:

18 $$\begin{aligned} \rho _{m n}=\frac{1}{4} \begin{pmatrix} \langle \Gamma _{m}^{\uparrow } \Gamma _{n}^{\uparrow }\rangle &{} \langle \Gamma _{m}^{\uparrow } \sigma _{n}^{-}\rangle &{} \langle \sigma _{m}^{-} \Gamma _{n}^{\uparrow }\rangle &{} \langle \sigma _{m}^{-} \sigma _{n}^{-}\rangle \\ \langle \Gamma _{m}^{\uparrow } \sigma _{n}^{+}\rangle &{} \langle \Gamma _{m}^{\uparrow } \Gamma _{n}^{\downarrow }\rangle &{} \langle \sigma _{m}^{-} \sigma _{n}^{+}\rangle &{} \langle \sigma _{m}^{-} \Gamma _{n}^{\downarrow }\rangle \\ \langle \sigma _{m}^{+} \Gamma _{n}^{\uparrow }\rangle &{} \langle \sigma _{m}^{+} \sigma _{n}^{-}\rangle &{} \langle \Gamma _{m}^{\downarrow } \Gamma _{n}^{\uparrow }\rangle &{} \langle \Gamma _{m}^{\downarrow } \sigma _{n}^{-}\rangle \\ \langle \sigma _{m}^{+} \sigma _{n}^{+}\rangle &{} \langle \sigma _{m}^{+} \Gamma _{n}^{\downarrow }\rangle &{} \langle \Gamma _{m}^{\downarrow } \sigma _{n}^{+}\rangle &{} \langle \Gamma _{m}^{\downarrow } \Gamma _{n}^{\downarrow }\rangle , \end{pmatrix} \end{aligned}$$

where, $\Gamma _{i}^{\uparrow }=\mathbbm {1}+\sigma ^z$ and $\Gamma _{i}^{\downarrow }=\mathbbm {1}-\sigma ^z$. Moreover, $\sigma ^{\pm }=\sigma ^{x}\pm i\sigma ^{y}$. For calculating concurrence, firstly we create the “spin-flipped” density matrix ${\tilde{\rho }}_{mn} \equiv \left( \sigma ^{y} \otimes \sigma ^{y}\right) \rho _{mn}^*\left( \sigma ^{y} \otimes \sigma ^{y}\right)$, in which $\rho _{mn}^*$ is the complex conjugate of the reduced density matrix. In Eq. (18), $\langle ~~\rangle$ indicates the expectation values of the specified operators over the quantum state defined in Eq. (15).

Then, for the operator $\rho _{m n} {\tilde{\rho }}_{m n}$, find the eigenvalues $\lambda _i$. Ultimately, the concurrence can be expressed as

19 $$\begin{aligned} C_{m,n}=\max \left\{ \sqrt{\lambda }_{1}-\sqrt{\lambda }_{2}-\sqrt{\lambda }_{3}-\sqrt{\lambda }_{4}, 0\right\} , \end{aligned}$$

where the eigenvalues $\lambda _i$s are ordered decreasingly. The concurrence ranges between 0 for an unentangled state to unity for a maximally entangled state. In what follows, we present the results of Eq. (19) for any pair of the chain in several situations. Because the quantum state changes over time, the density matrix and consequently concurrences are varied by time. For the considered system, using Eq. (15), concurrence between nearest neighbors is obtained as the following closed form:

20 $$\begin{aligned} C_{m,m+1}=\sqrt{\mathscr {C}_m\mathscr {C}_{m+1}}~~, \end{aligned}$$

where

21 $$\begin{aligned} \mathscr {C}_m=\left| \mathcal {J}_{m-1}({\tilde{J}} t) \right| ^2+\left| \mathcal {J}_{m-2}({\tilde{J}} t) \right| ^2-2~\mathcal {J}_{m-1}({\tilde{J}} t)\mathcal {J}_{m-2}({\tilde{J}} t) \sin (\phi +\alpha ), \end{aligned}$$

and $\mathscr {C}_{m+1}$ can be generalized from Eq. (21). This relation is comparable to the very familiar relation of wave interference^28^, except that instead of the interfering waves’ amplitudes, Bessel functions are here taken into account. In Eq. (21), $\sin (\alpha +\phi )$ is a crucial and significant function which determines how entanglement propagates across the chain as we will see in the following section. In this equation, the sign of the last term determines the quality of entanglement transmission. In this regard, if $\sin (\phi +\alpha )<0$, the last term of the above relation become positive around the first maximum of Bessel functions. This condition signifies the constructive interference in wave-interference language. On the other hand, in the situation where $\sin (\phi +\alpha )>0$, depending on the sign of the Bessel functions, in some parameter ranges, the speed of entanglement transmission decreases. This situation signals out the destructive interference in wave-interference language. Furthermore the maximum amount of concurrence that receives any pair reduces in this circumstance. These subjects will be covered in further details over sections that follow. In the frame of time evolution of concurrence [Eqs. (20) and (21)] we can access the details of entanglement propagation such as the speed of spreading or the dependence of entanglement transmission on the phase factor. We will deal with details in the next section.

**Figure 1:**
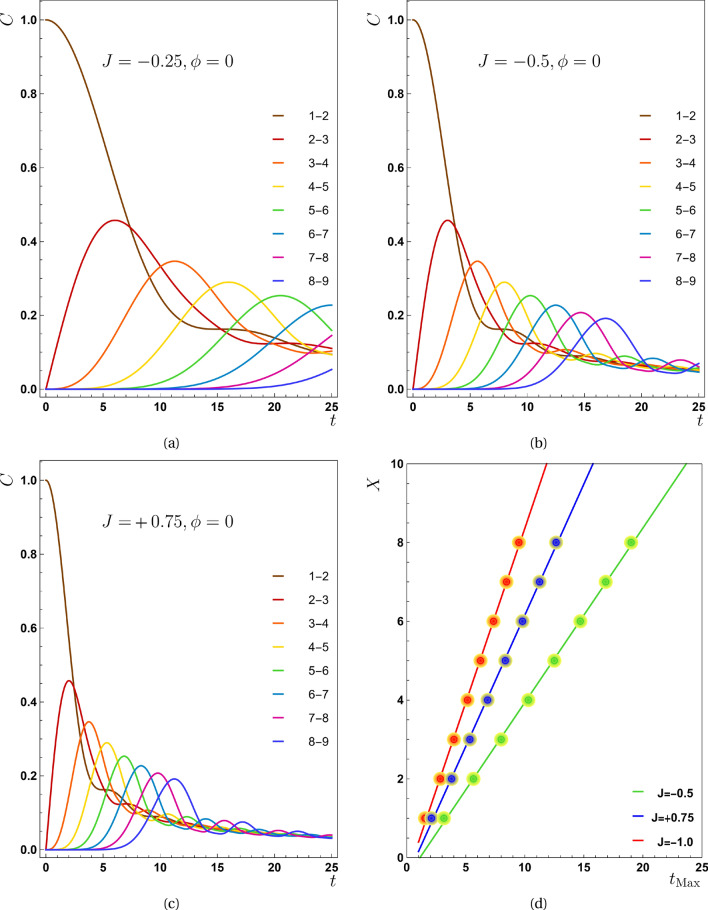
In the first scenario ($J\ne 0,D=0$) the concurrence as a function of time *t* (**a**) $J=-0.25$, (**b**) $J=-0.5$, (**c**) $J=0.75$. As can be seen, the maximum value of the concurrence function for the various pairs occurs in the shorter time instances as J increases, and the graphs become more compact as a result. The diagram of $X-t_{Max}$ for different values of exchange interaction is presented in panel (**d**), from which the value of propagation speed for $J=-0.5$ is $0.4410\pm 0.0005$, for $J=0.75$ and $J=-1.0$, this quantity equals with $0.6620 \pm 0.0005$ and $0.8830 \pm 0.0005$ respectively and for all plots $\phi =0$.

**Figure 2:**
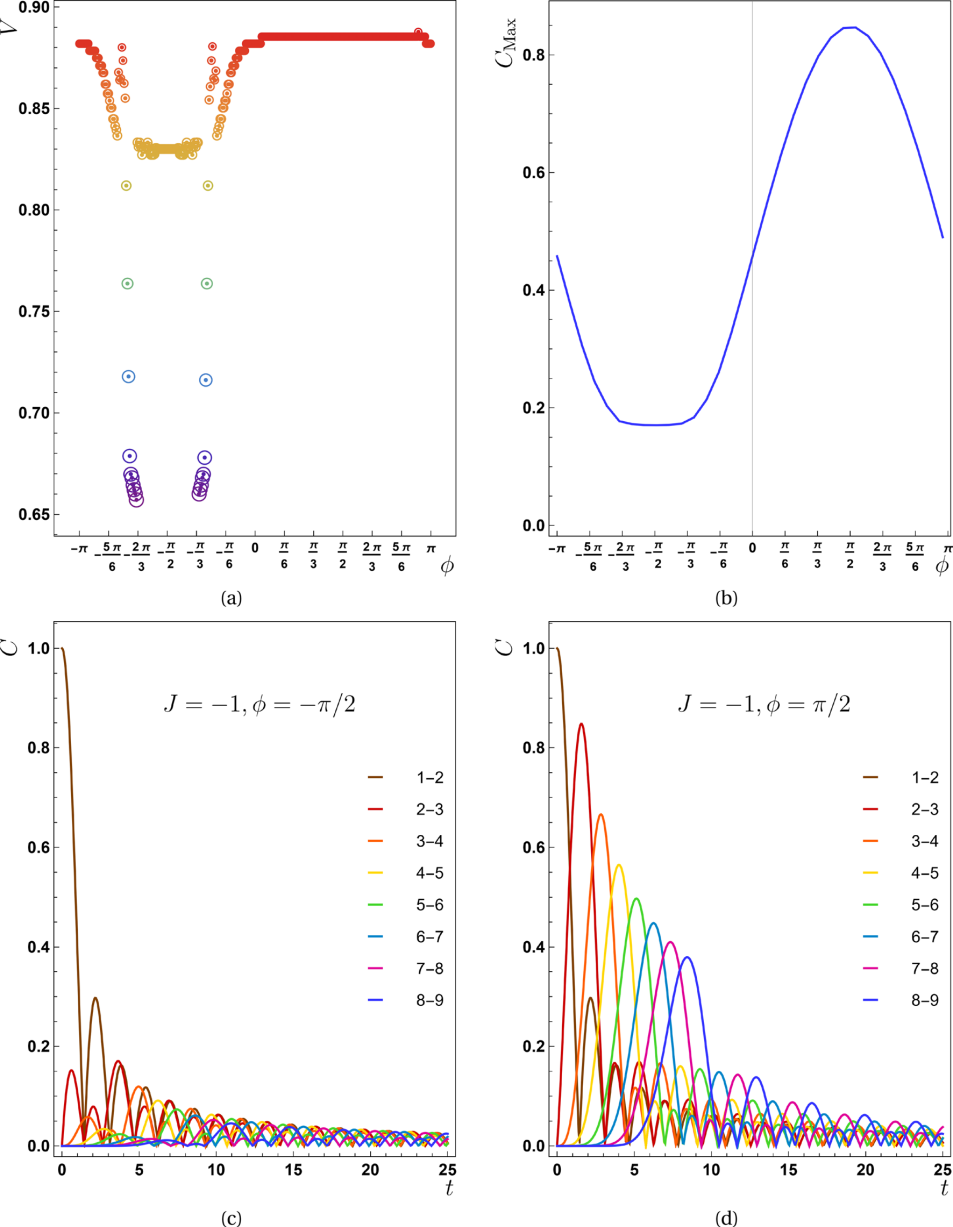
(**a**) The propagation speed is plotted against the phase factor ($\phi$) with $J=-1.0$. The drops that experienced by this quantity is around $\phi =\pi /2$. (**b**) The maximum value of the concurrence function for the pair (2, 3) versus $\phi$. By comparing panels (**a**) and (**b**), we can see that the lowest entanglement value is responsible for a phase interval during which the velocity reaches its lowest value. Panels (**c**) for $\phi =-\pi /2$ and (**d**) for $\phi =\pi /2$ show the effect of $\phi$ on the maximum entanglement. The last two panels are in good agreement with the panel (**b**).

## Results

Using Eq. (20), we outline the details of the entanglement transfer process in the XX-spin chain modulated by the DM interaction. We suggest three scenarios: first, we evaluate the transfer process without DM interaction, then we examine the procedure with just this potential, and eventually we analyze the condition during which both types of couplings coexist.

To implement the first scenario, in the absence of DM interaction, we substitute $\alpha =\pi$ and $\alpha =0$ for $J< 0$ and $J> 0$ respectively and ${\tilde{J}}=\left| J\right|$ in Eq. (15). Figure 1a–c demonstrate the behavior of concurrence versus time for different values of *J* and $\phi =0$. Each curve in these plots is related to one pair of qubits. According to the plots, each pair of particles in the chain reaches a maximum amount of entanglement over time. The farther a pair is from the initially entangled one (IEP), the longer it takes to reach maximum entanglement which itself decreases with distance from the IEP. Comparing Figs. 1a–c clearly shows that when the Heisenberg interaction increases, the curves of concurrences get more compact, implying that the concurrence’s maxima occur in a shorter period of time. This behavior is the same for positive and negative values of *J*.

In the following, we use the symbol *X* to show the distance between a typical neighbor $(m,m+1)$ and the IEP. For example, this amount equals unity in the case of the pair (2, 3). In the next step, we determine the time instances ($t_{Max}$) associated with entanglement peaks for every *X*. So in Fig. 1d, this parameter is drawn against these instances for different values of *J*. The slope of these functions defines the entanglement propagation speed (*V*). When we use the letter *V*, we are referring to the magnitude of velocity which is known as speed in physics. The curves of Fig. 1d demonstrate that raising the amount of *J* increases propagation speed, linearly, i.e., doubling the *J* value does double the speed value.

Another crucial point to consider here is whether the phase factor of the initial state [Eq. (7)] ($\phi$) influences entanglement transmission behavior!? This parameter was first introduced in Eq. (7) and its significance for the concurrence function is provided in Eq. (21). Figure 2 depict the mysterious function of this parameter in the process of entanglement propagation. At this stage, $\phi$ was tuned very slightly and correspondingly *V* was evaluated for each value of $\phi$. Panel (a) shows how speed is affected by this parameter. As can be seen in this plot, *V* decreases dramatically at several values of the phase factor in the region of $-\pi<\phi < 0$. The values of $\alpha$ is equal to $\pi$ because the parameters chosen for this shape are known to be $J=-1$ and $D=0$. As previously mentioned, the term containing the sine function is positive in Eq. (21) for this interval of $\phi$, but when looking at the sign of this term, it plays a decreasing role over the whole $\mathscr {C}_m$ function. For this reason, we observe a sharp reduction in both the speed function and the maximum of entanglement for this interval of $\phi$.

Panel (b) of Fig. 2 depicts the maximum entanglement of pair (2, 3) for different values of $\phi$. Because the peaks of concurrence for all pairs have similar behavior, the vertical line is nominated by $C_{Max}$. A comparison of panels (a) and (b) reveals that the speed profile drops at special values of $\phi$ that followed by decreasing $C_{Max}$ as well. Of course, this is neither generalizable nor obvious because for a region of $\phi$ in which a greater quantity of $C_{Max}$ reaches various pairs, *V* does not abruptly rise. This fact is deduced by comparing the flat region of panel (a) to the peak of $C_{Max}$ in the panel (b) for $0<\phi \le \pi$. To support our assertions, we displayed in panel (c) and (d), the concurrence function over time for $J=-1$ and $\phi =\{\pi /2,-\pi /2\}$. As it is clear in these plots $C_{Max}$ (for all pairs) at $\phi =-\pi /2$ is less than the same function for $\phi =\pi /2$.

**Figure 3:**
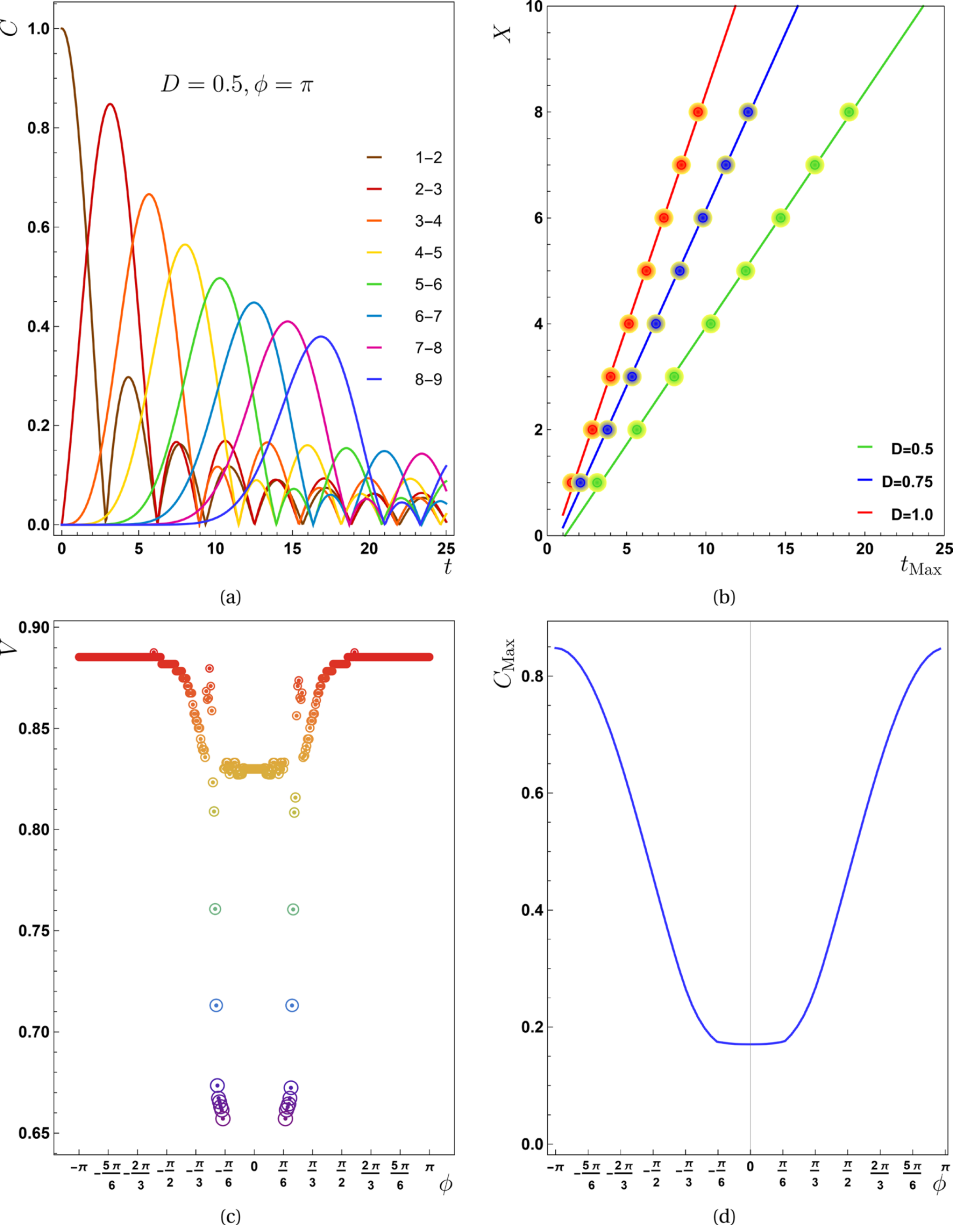
In the second scenario ($J=0,D\ne 0$) (**a**) the concurrence as a function of time *t* for $D=0.5$ is presented. (**b**) The diagram of $X-t_{Max}$ for different values of *D* is shown in this panel from which, the value of propagation speed for $D=0.5$, $D=0.75$ and $D=1.0$ is obtained as $0.4435 \pm 0.0005$, $0.6664 \pm 0.0005$ and $0.8854 \pm 0.0005$ respectively. (**c**) The propagation speed with respect to the phase factor ($\phi$) is represented here for $D=1$. The drops that experienced by this quantity is around $\pi /2$ which differs from previous case (the first scenario). (**d**) The maximum value of the concurrence function for the pair (2, 3) versus $\phi$. By comparison between panels (**c**) and (**d**) we can see that the lowest value of entanglement is responsible for an interval of phase, for which the speed reaches its lowest value.

**Figure 4:**
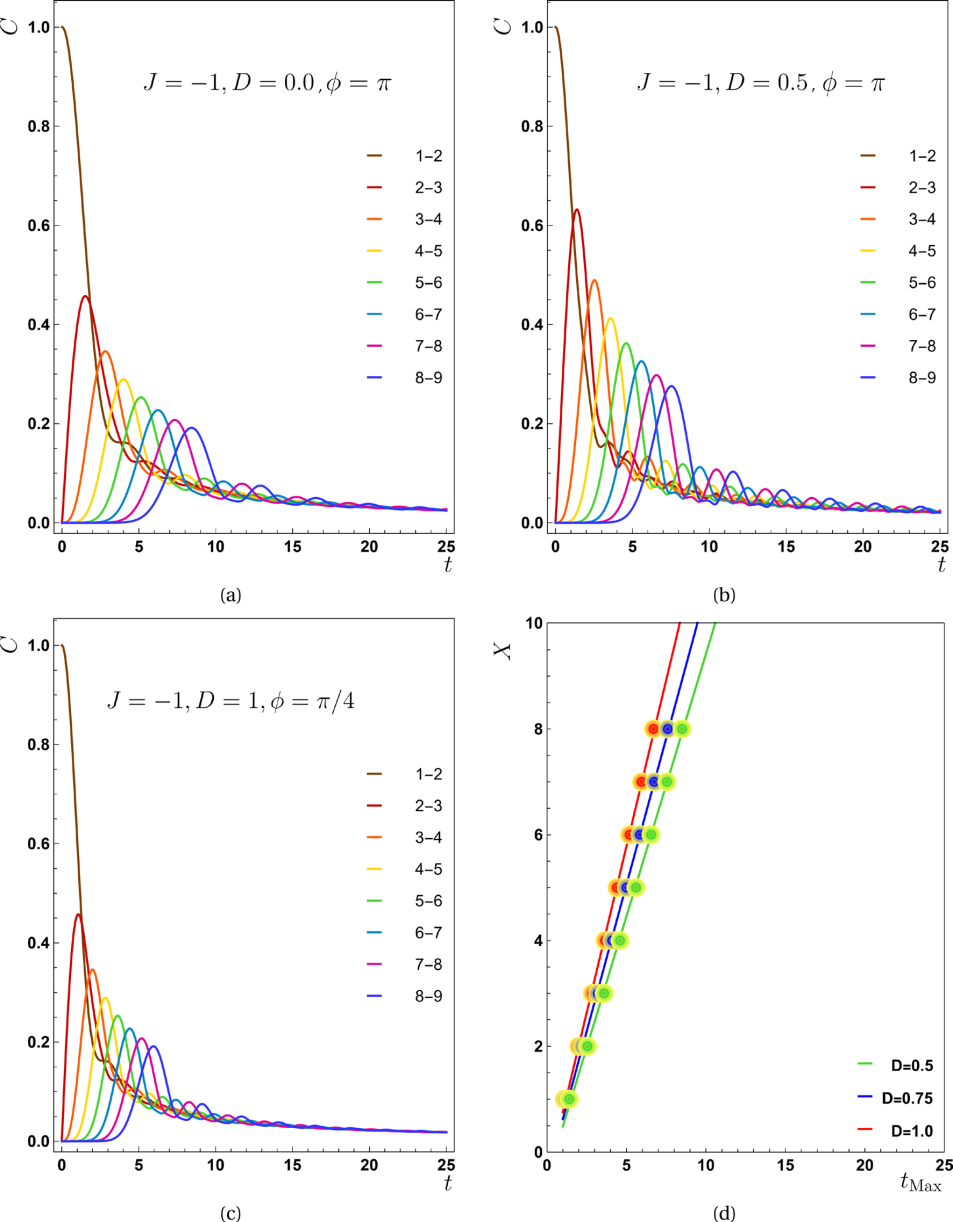
In the third scenario ($J<0,D\ne 0$) the concurrence as a function of time *t* (**a**) $D=0.0, \phi =\pi$, (**b**) $D=0.5, \phi =\pi$, (**c**) $D=1.0, \phi =\pi /4$ and for all plots $J=-1.0$. As *D* increases, the maximum value of the concurrence function for the various pairs occurs in shorter time instances indicating *V* grows, however, the maxima of concurrences do not grow (the details are explained in Fig. 5**b**). Panel (**d**) shows the $X-t_{Max}$ diagram for various values of *D*, from which the propagation speed for $D=0.5$ is $0.9930\pm 0.0005$, for $D=0.75$ and $D=1.0$, this quantity equals with $1.1070 \pm 0.0005$ and $1.2570 \pm 0.0005$ respectively.

Ultimately, following is a summary of the subject matter concerning the effects of *J* on the transition process: we find that increasing the Heisenberg strength interaction leads the propagation speed to grow linearly. Furthermore, the effect of $\phi$ on the speed profile is asymmetric, resulting in a sharp decrease in *V* across a range of phase factors. Corresponding to that range of $\phi$, we have a decline in $C_{Max}$ received by each pair. Additionally, as shown in Section “Other contradictions in the first scenario” the maximum amount of entanglement that may be transferred from the IEP to any certain pair is not affected by increasing the value of *J*.

At the next step (before we examine the influence of the DM interaction in the presence of Heisenberg coupling), we study only the effect of the DM interaction on the speed and amount of entanglement received by pairs of the chain. For this purpose, based on $\alpha =\pi /2$ we modify Eq. (15) for $|\psi (t)\rangle$ as:

22 $$\begin{aligned} |\psi (t)\rangle =\frac{1}{\sqrt{2}}\sum _{j}e^{-i\pi (j-1)}\Big [\mathcal {J}_{j-1}(tD) + e^{i(\phi +\pi )} \mathcal {J}_{j-2}(tD) \Big ]c^\dagger _j|00..0\rangle . \end{aligned}$$

The group of Fig. 3 may help us understand the distinction between the circumstances where we only have *D* and those where we only have *J*. Panel (a) shows the behavior of the concurrence function in terms of time for each pair of the chain. Selected values for extracting this panel are $D=0.5$ and $\phi =\pi$; by this value of $\phi$ we are away from the reduction region ($-\pi /2<\phi <\pi /2$ that is determined in panel c). Other results that were obtained for different values of *D* and the same value of $\phi$ are not directly presented here. Similar to the preceding scenario, the value of *V* is determined for various values of *D* after finding the *X* parameter and $t_{Max}$ for each pair (panel b). By adjusting $\phi$ and calculating *V* accordingly, we arrive at panel (c). Then the effect of phase factor on the speed as well as the maximum amount of entanglement is presented in Fig. 3c and d respectively. The same sudden drop in speed profile generated by some phases is shown here, however the interval of such a collapse differs from the prior example (only *J*). This period refers to the interval during which the highest entanglement fell to their lowest values (panel d). Comparing Figs. 2a and 3c shows that the maximum amount of *V* is the same for both of scenarios, however they occur in different values of $\phi$ parameter as we expected.

**Figure 5:**
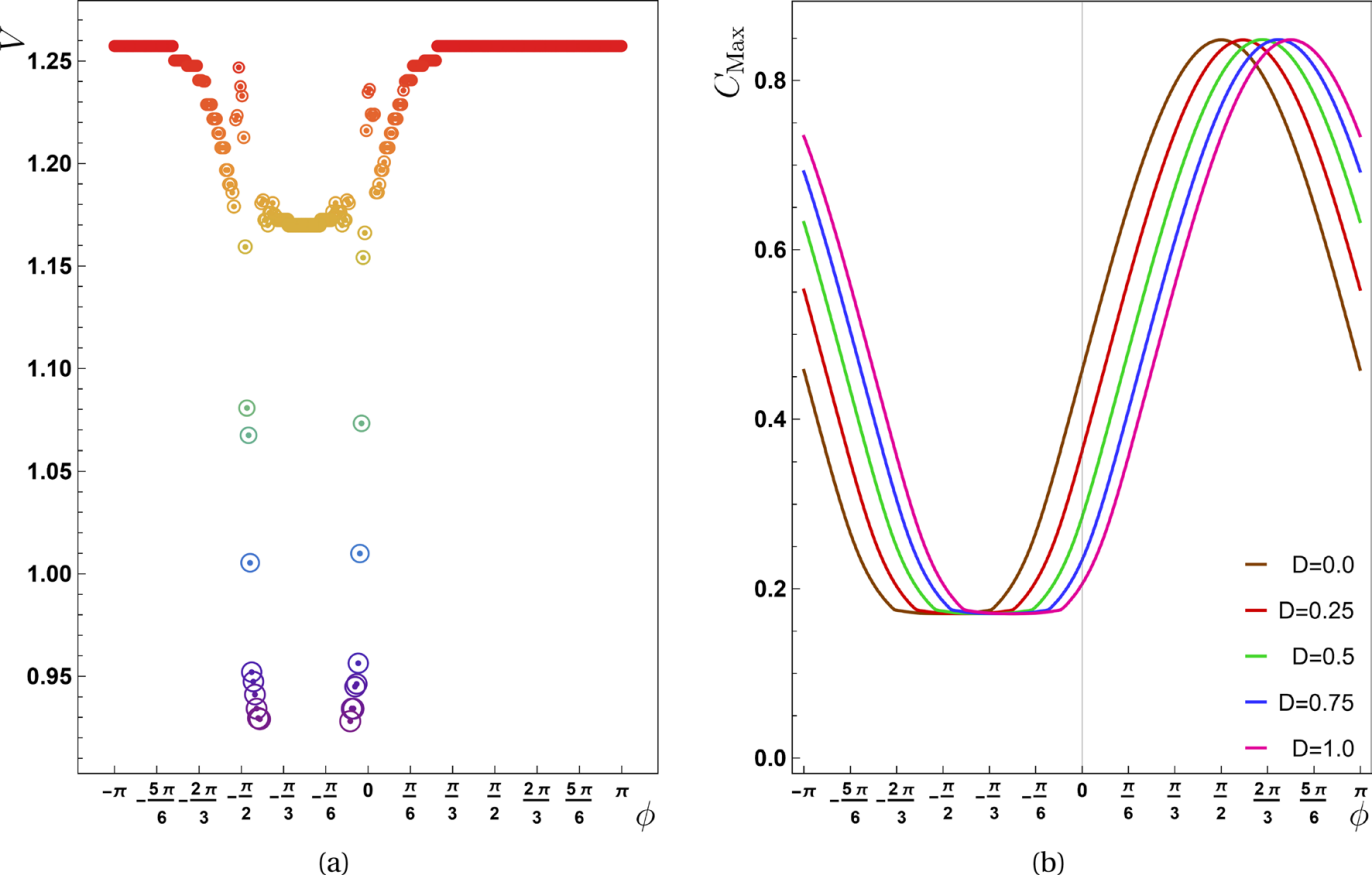
For the third scenario: (**a**) the propagation speed in relation to the phase factor ($\phi$), and (**b**) the maximum value of the concurrence function for the pair (2, 3) versus $\phi$.

The coexistence of *D* and *J* produces intriguing effects, as if a combination of two preceding situations may be seen here. Images shown in Fig. 4 depict the transmission of entanglement for the present scenario. For drawing these plots, we set $J=-1$, $\phi =\{\pi ,\pi /4\}$ and *D* to be varied so that we can compare the results with Ref.^24^. Panels (a)–(c) also show that when the value of *D* grows, the curves of concurrence get more compact, implying that raising the value of *D* causes the entanglement to propagate faster. Panel (d) is designed to demonstrate how the transfer speed rises with increasing values of *D*, and the numerical findings are presented in these figures’ caption that are in well agreement with Ref.^24^. Additionally, as demonstrated by comparing panels (a)–(c), an increase in the value of *D* alters the maximum entanglement that each pair can achieve. The plots illustrate we cannot conclude that $C_{Max}$ will always expand as *D* does. In fact, the value of $C_{Max}$ is highly dependent on the value of $\phi$, so increasing the value of *D* either improves or impair it. This dependency is clarified by Fig. 5b which will be discussed in details in its place. This result contradicts Ref.^24^ where rising *D* has no effect on the maximum entanglement transmitted in the presence of *J*.

Nevertheless, owing to the ${\tilde{J}}$ growth, raising *D* always leads to a greater speed. In this scenario, however, unlike the previous two, the process is not linear, i.e., a twofold increase in *D* will not result in a twofold increase in speed.

The second dissent between our work and Ref.^24^ concerns the speed’s dependency on the phase factor. For a range of this parameter, there is a drop in the speed profile as seen in Fig. 5a. Here, using $J=-1$ and $D>0$, it is found that $\cos (\alpha )=J/{\tilde{J}}<0$ and $\sin (\alpha )=D/{\tilde{J}}>0$, indicating $\alpha$ is in the second quadrant of the trigonometric circle. Therefore, $\sin (\phi +\alpha )<0$ if $0<\phi <\pi$. Since the destructive interference is responsible for the drops in the speed profile, and it happens when the last term of Eq. (21) becomes negative when $\sin (\phi +\alpha )>0$, then the drop region does not happen for $0<\phi <\pi$. A similar result can be obtained for $J>0$, because in this situation, $\alpha$ is in the first quadrant of the trigonometric circle and $\sin (\phi +\alpha )<0$ if $0<\phi <\pi$. Although similar behavior existed in all scenarios, the range of phase factor for sudden reduction differs between them.

Finally, in the second panel of Fig. 5, the $C_{Max}$ function is drawn in terms of $\phi$. As it is clear, the minimum of this function corresponds to the drop area of the first panel. The dependency of $\alpha$ on the value of *D* makes the minimum position of $C_{Max}$ changes as the value of *D* increases. In fact, the curve by $D = 0$ has been also displayed in the first scenario; but for $|J|\ll D$, the minimum of this function experiences $\phi =0$ as shown in the second scenario. An important note to be marked is related to the behavior of $C_{Max}$ towards increasing *D*. This conduct strongly dependents on the value of $\phi$. For example by choosing $\phi =\pi /4$, $C_{Max}$ reduces as *D* grows. On the other hand, for $\phi =\pi$, this function rises as *D* grows. This result is in well agreement with Fig. 4a–c.

**Figure 6:**
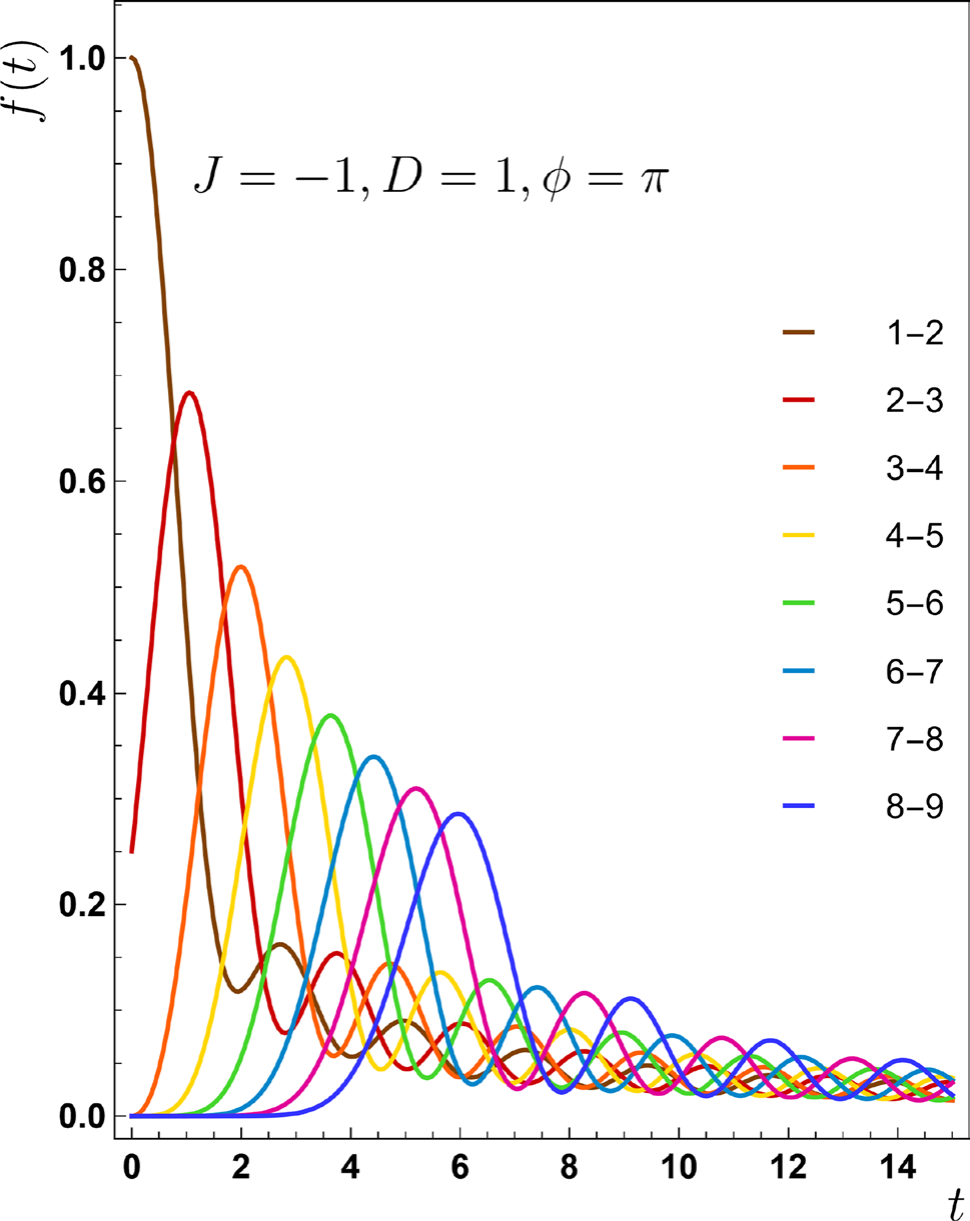
Fidelity for various pairs to ensure that entanglement transmission is according to QST.

Here is a quick explanation of why it is reasonable to believe that entanglement transmission and QST are close relatives. The fidelity function [Eq. (23)] is an easy way for measuring the similarity between two quantum states, particularly when the input state is pure^29^:

23 $$\begin{aligned} f(t)=\langle \psi _{\text {int}}|\rho _{\text{ out } }|\psi _{\text {int}}\rangle . \end{aligned}$$

This function is unity when $\rho _{\text{ out }}$ equals with $\rho _{\text{ int } }=|\psi _{\text {int}}\rangle \langle \psi _{\text {int}}|$. On the other hand, $F=0$ indicates that output state is completely orthogonal to the input one. It is worth to notice that $|\psi _{\text {int}}\rangle$ is not the initial state [Eq. (7)] but is defined as $(c_j^\dagger +e^{i\phi }c_{j+1}^\dagger )|0\rangle$. By this interpretation, fidelity somehow shows how much a received quantum state by pair $(j,j+1)$ is similar to a completely entangled state between them. Then it indicates the similarity of transferred state and initial entangled one. In addition, $\rho _{\text{ out }}$ is available from Eq. (18), then we can access to the fidelity’s closed form as

24 $$\begin{aligned} f(t)=\frac{1}{2}|e^{i\phi }a_{j}(t)+a_{j+1}(t)|^2, \end{aligned}$$

where $a_{j}(t)$s are the coefficients defined in Eq. (15). Figure 6 shows how fidelity behaves for the various pairs of the chain. By consideration on this plot, it appears another controversy between our results and ones presented for this function in Ref.^24^, because, it is reasonable that fidelity starts from 0.25 for pair (2, 3). At $t=0$, $a_2=1/\sqrt{2}$ and $a_3=0$, then inserting them in Eq. (24) results in $F=0.25$, opposite to which is presented in that reference.

**Figure 7:**
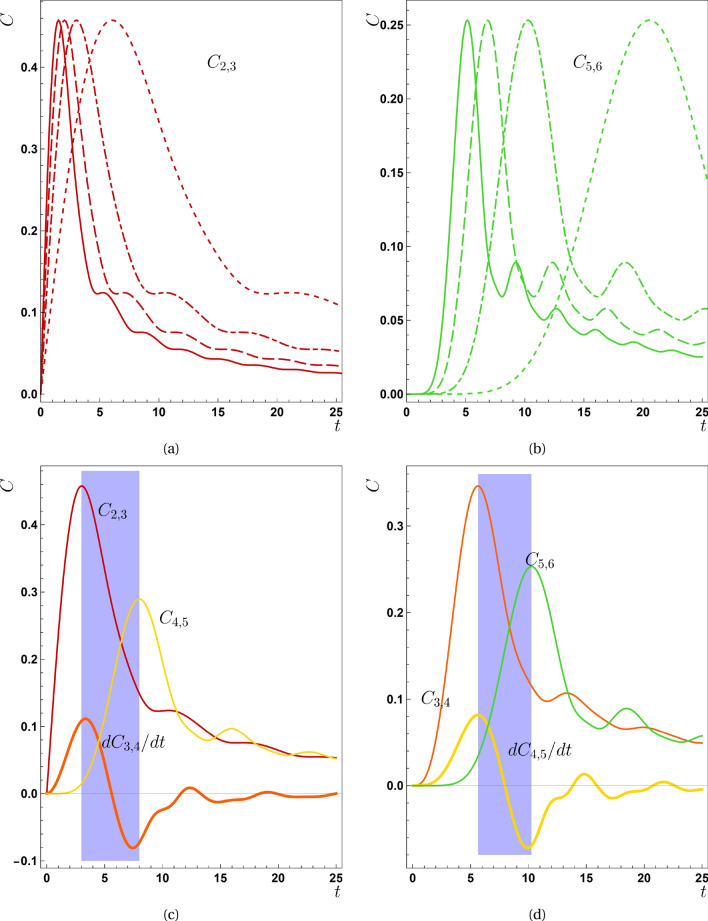
The concurrence-time function for an individual pair (**a**) (2, 3) and (**b**) (5, 6). In fact these plots shows the dependency of maximum entanglement on *J* values shared in discrete pairs. In both of plots dotted lines corresponds to $J=-0.25$, dotdash lines addresses $J=-0.5$, dashed lines matches to $J=-0.75$ and finally solid lines is associated with $J=-1.0$. The maximum entanglement for all *J* values is the same for pairs (2, 3) in panel (**a**). This rule also holds true for pair (5, 6) in panel (**b**). This phenomenon is generalizable for other pairs. (**c**) The time derivative of concurrence $(\frac{\partial C_{m,m+1}}{\partial t})$ for pair of (3, 4) and (**d**) for pair of (5, 6). They are representative other pairs and mimic their behaviors.

## Other contradictions in the first scenario

As mentioned in the first scenario, examining topics such as the change of the maximum value of entanglement for a specific pair with respect to the changes of *J* and also the derivatives related to the concurrence function are assigned to this section. As it is obvious from Fig. 7a and b the maximum amount of entanglement that reaches any pairs of the chain does not change as *J* increases, while the propagation speed does. For example, for the pair (2, 3), this amount is near to 0.46, and for the pair of (5, 6), it is close to 0.25 for all values of *J* (see Fig. 7a,b). This phenomenon takes place for the second scenario in which there is just DM interaction. But in the third proposition, $C_{Max}$ varied as magnetic interaction changes; that its reason is related to the dependency of $\alpha$ on the magnetic interaction.

In Fig. 7c and d the time derivative of $C_{3,4}$, and $C_{4,5}$ is presented respectively. They are representative of other pairs and mimic their behaviors i.e., we can generalize their behaviors to other pairs. We can see from these plots that $\left| (\frac{\partial C_{m,m+1}}{\partial t})\right|$ approximately (not exactly) reaches its maximum value when $C_{m-1,m}$ or $C_{m+1,m+2}$ is at their maximum values. This is also the point where our findings do not fully agree with the results of Ref.^24^ that brings up the maximum value of $C_{m-1,m}$ or $C_{m+1,m+2}$ does exactly occur in the extrema of $\left| (\frac{\partial C_{m,m+1}}{\partial t})\right|$. Our findings shows when the concurrence shared on $(m-1,m)$ reaches approximately its maximum value, it transferred to the next pair with the highest rate. In addition, when the pair $(m+1,m+2)$ is to be at the maximum amount of entanglement, the reduction rate in the previous pair $(m,m+1)$ almost is at the maximum. We further examine here why we utilized absolute maximums rather than relative extrema in the concurrence function. By consideration on Fig. 7, we can observe that if entanglement is to reach a pair from the previous neighbor and then be transmitted to the next neighbor at a substantial rate, it must occur around the absolute maximum rather than near the relative maxima. This fact holds true for all scenarios.

## Conclusion

One-dimensional spin chains have attracted researchers’ attention for their QST features, which has led to a focus on the implications of such systems. The synergy between the quantum information community and the realm of quantum magnetism opens up a world of possibilities, such as using spin structures in entanglement transmission as an example of QST process. The present work has dwelt on QST aspects of 1D spin-1/2 XX model with DM interaction. We considered an initial quantum state for the system in which entanglement is shared between just two first spins and other particles are disentangled. The time-dependent quantum state and reduced density matrix for any nearest neighbors spins were produced as a function of the DM interaction and Heisenberg coupling using the time evolution operator. Then, to analyze entanglement transmission throughout this system, we devised three scenarios: (i) in the absence of DM interaction, (ii) in the absence of Heisenberg interaction, and (iii) coexistence of both types of couplings.

At the first step, we derived an exact solution for time-evolved wave function of the system by Jordan–Wigner transformation. Then, we drew the concurrence function (as measure of entanglement) in terms of time for each pair of nearest neighbors separated by *X* from the IEP (that is abbreviated of initial entangled pair). We discovered that the time required for the greatest amount of entanglement to reach a pair ($t_{Max}$) at a distance *X* is proportional to *X*. Then, we calculated the numerical value of speed of entanglement propagation from the slope of $X-t_{Max}$ diagram. The speed was found to be linearly related to the strength of magnetic interactions in the first and second scenarios, but not in the third.

We also looked at how the phase factor (represented by $\phi$) that controls the system’s initial state impacts entanglement propagation. It is revealed that the speed of entanglement propagation drops dramatically with certain phase factors. This is a characteristic that occurs in all contexts; however, the phase interval during which sharp drops are recognized, varies depending on the conditions. We have also shown how to predict the location of these dramatic drops using the language of wave interference.

It was discovered that the maximum entanglement (that symbolized by $C_{Max}$), which plays an essential role in calculating the value of transmission speed, is likewise a function of $\phi$. This function has a minimum in the phases that are responsible for the drops in speed profile. This function was unaffected by the modification of the amount of magnetic interaction in either of the two first scenarios. While, altering the magnitude of the magnetic interaction in the third scenario did have an effect on $C_{Max}$’s dependence on $\phi$. The consequence of such an effect is that, as the amount of magnetic interaction increases, not only does the speed increase, but the $C_{Max}$ also becomes a function of the amount of interaction; this situation was the result of the coexistence of both interactions. This problem was in contradiction with Ref.^24^, whereas we are in good line with that reference in the retrieved numerical values for speed.

## Data Availability

The data-sets used and analyzed during the current study available from the corresponding author on reasonable request.
